# Vitamin D and chronic kidney disease: an uneasy relationship

**DOI:** 10.1590/2175-8239-JBN-2020-E002

**Published:** 2020-11-30

**Authors:** Fellype Carvalho Barreto, Andréa Emilia Marques Stinghen

**Affiliations:** 1 Universidade Federal do Paraná, Departamento de Clínica Médica, Curitiba, PR, Brasil.; 2 Universidade Federal do Paraná, Laboratório de Nefrologia Experimental, Departamento de Patologia Básica, Curitiba, PR, Brasil.

Over the last years, vitamin D has gained the status of a multi-hormone with functions far beyond its calciotropic actions. Several experimental and clinical studies have supported that vitamin D may play a role on endothelial function and the immune system. Furthermore, vitamin D deficiency has been associated with a wide variety of acute and chronic illnesses, such as infectious, autoimmune, and cardiovascular diseases, stroke, type 2 diabetes, and others^1^. In the context of chronic kidney disease (CKD), it is recognized that vitamin D deficiency is highly prevalent and it has been associated with left ventricular hypertrophy and increased mortality independently of vascular calcification and stiffness^2^^,^ 3. Moreover, hypovitaminosis D in hemodialysis patients has been linked to an increased number of circulating intermediate monocytes and decreased classical monocytes, suggesting that low vitamin D levels might contribute to the inflammatory profile found in these patients by modulating the subtype of monocyte population^4^.

In the study published in this issue of the *BJN,* Matsumoto *et al*. investigated the association between deficiency of 25-hydroxyvitamin D and inflammatory and oxidative stress in a cohort of pre-dialysis CKD patients^5^. The study included 206 pre-dialysis CKD patients who were not using vitamin D supplementation. A large number of inflammatory and oxidative stress biomarkers, such as interleukin (IL)-6, adiponectin, F2-isoprostane, advanced oxidation protein products (AOPP), hs-C reactive protein (hs-CRP), were measured. The prevalence of vitamin D deficiency, defined as serum levels of 25(OH)-vitamin D below 20 ng/mL, in the study population was 27% (55/204) and it was inversely correlated with renal function. The multivariate analyses could not demonstrate any significant effect of vitamin D on the levels of inflammatory and oxidative stress biomarkers^5^. Contrarily, CKD stages were correlated with oxidative stress. The authors concluded that vitamin D deficiency might not play a role in the increased oxidative stress state commonly seen in pre-dialysis CKD patients^5^.

The finding of Matsumoto *et al.* adds new fuel to the debate about the role of vitamin D in contributing to the CKD-related inflammatory environment. Indeed, uremia is characterized by the accumulation of a myriad of uremic toxins and metabolic derangements that may induce inflammation and oxidative stress. Therefore, it could be somewhat expected that (i) the superiority of one marker, in this case serum levels of vitamin D, over the others could not be demonstrated and that (ii) renal function, as a global marker of uremia, would explain the greater levels of inflammatory and oxidative stress biomarkers seen in these patients. However, the small sample size, stated as a study limitation by the authors^5^, may have precluded a more robust statistical analysis.

Furthermore, despite of the growing evidence grounded on experimental and observational studies of the pleiotropic actions of vitamin D, it has not been an easy task to prove that vitamin D supplementation may have beneficial effects *per se*. In a large, randomized, placebo-controlled trial, supplementation with vitamin D at a daily dose of 2000 IU together with omega-3 fatty acids was not capable of lowering the risk of cardiovascular events among men 50 years of age or older and women 55 years of age or older^6^. Our group has investigated the role of vitamin D supplementation (cholecalciferol) on the inflammatory profile of CKD patients with hypovitaminosis D in hemodialysis. Even though cholecalciferol presented anti-inflammatory effects *in vitro*, the 6-month supplementation restored the vitamin D levels but was not effective in improving the inflammatory profile, as measured by IL-1β and hs-CRP, in the clinical setting (personal data). Otherwise, others have reported a beneficial role of vitamin D on inflammation and left ventricular hypertrophy in CKD patients^7^.

The actions of vitamin D beyond mineral metabolism are undeniable. However, uremia negatively affects body functions through a multitude of factors, such as anemia, uremic toxins accumulation, hypervolemia, among others. Therefore, not finding an association between vitamin D levels and biomarkers of inflammation and oxidative stress is not necessarily a proof that vitamin D does not play a role on these pathways. Above all, the study of Matsumoto *et al*. alerts us of the complexity of chronic kidney disease and, in a broader view, the importance of preserving renal function.
